# The TET Protein Family in Gliomas: Epigenetic Regulation, Molecular Pathogenesis, and Therapeutic Opportunities

**DOI:** 10.3390/life16101672

**Published:** 2026-10-07

**Authors:** Angelos Kandilas, Dionisis Leimonis, Ioannis Vasilakis, Elisavet Kosma, Christina Piperi

**Affiliations:** Department of Biological Chemistry, School of Medicine, National and Kapodistrian University of Athens, 11527 Athens, Greece; a.kandilas7@gmail.com (A.K.); leimonisdionisis@gmail.com (D.L.); jv10lm@gmail.com (I.V.); elisavet3kosma@gmail.com (E.K.)

**Keywords:** brain tumors, TET proteins, 5hmC, DNA methylation, IDH mutation, epigenetic therapy

## Abstract

Epigenetic dysregulation is a defining feature of glioma pathogenesis, with aberrant DNA methylation contributing to tumor initiation, cellular plasticity, progression, and therapeutic resistance. The ten-eleven translocation (TET) family of dioxygenases (TET1, TET2, and TET3) catalyzes the 5-methylcytosine (5mC) oxidation to 5-hydroxymethylcytosine (5hmC) and subsequent intermediates, inducingactive DNA demethylation and regulating organization of chromatin and gene expression. In gliomas, particularly those harboring isocitrate dehydrogenase (IDH) mutations, accumulation of the 2-hydroxyglutarate oncometabolite suppresses TET activity, leading to widespread epigenetic remodeling, the glioma CpG island methylator phenotype and impaired cellular differentiation. Emerging evidence demonstrates that individual TET isoforms exert distinct and context-dependent biological functions. Although TET1, TET2, and TET3 have been involved in the regulation of glioma biology, their effects may vary depending on the cellular and molecular context, with individual isoforms exhibiting distinct and, in some cases, opposing effects on glioma behavior. This review summarizes current knowledge regarding the molecular mechanisms governing TET dysregulation in gliomas, including their interactions with metabolic alterations, chromatin remodeling, the tumor microenvironment, and immune evasion. Recent advances in methodologies for assessing TET activity and 5hmC levels are also discussed along with the possible integration of TET-associated biomarkers into molecular glioma classification, and emerging therapeutic strategies to restore or selectively modulate TET function.

## 1. Introduction

Gliomas present the most common type of malignant central nervous system (CNS) tumor in adults, comprising roughly 80% of the cases [1]. The WHO CNS5 classification divides CNS tumors into glial and glioneuronal/neuronal tumors according to their age of onset, cellular origin, and molecular profile [2]. CNS tumors range from lower-grade tumors, including certain types of astrocytomas and oligodendrogliomas, to highly aggressive glioblastomas (GB). Despite the progress of neurosurgery, chemotherapy and radiotherapy, the prognosis of GB remains poor [3]. Dismal prognosis is associated with the high genetic heterogeneity of the CNS tumors that affects epigenetic regulations and metabolic pathways involved in tumor aggressiveness [3,4].

Mutations in NADP(+)-dependent isocitrate dehydrogenase 1 (*IDH1*) and *IDH2* genes are among the most frequent genetic alterations in adult-type diffuse gliomas [5,6]. Also, it has been demonstrated that in IDH-mutant adult-type diffuse gliomas, the metabolite α-ketoglutarate (α-KG) is converted into 2-hydroxyglutarate (2-HG) and contributes to epigenetic dysregulation involved in gliomagenesis [5,7]. When 2-HG accumulates, it inhibits α-ketoglutarate-dependent dioxygenases, including histone demethylases and the Ten-Eleven Translocation (TET) family DNA hydroxylases affecting DNA and histone demethylation, respectively [4,5,7]. Consequently, inhibition of TET activity can impair the 5-methylcytosine (5mC) conversion into 5-hydroxymethylcytosine (5hmC) and contribute to DNA hypermethylation. This altered epigenetic landscape is associated with the CpG island methylator phenotype (G-CIMP), a hallmark of IDH-mutant gliomas, characterized by promoter hypermethylation and consequent gene silencing [3,8,9,10].

The TET protein family, composed of TET1, TET2 and TET3, is involved in the dynamic epigenetic modification of DNA methylation status [4]. TET1-3 use Fe(II) as a cofactor and α-KG as a co-substrate to catalyze the stepwise oxidation of 5mC to 5hmC, 5-formylcytosine (5fC) and 5-carboxylcytosine (5caC) in DNA [4,5]. In neural cells, TET-mediated DNA hydroxymethylation promotes an euchromatin state and controls the expression of genes involved in neuronal differentiation [4].

Hypermethylation of tumor-suppressor genes and de-methylation of oncogenes can result in tumor initiation, progression and metastasis [11]. There are significant in vitro studies indicating that in high-grade gliomas, both in IDH-wild-type and IDH-mutant, extensive loss of DNA 5hmC and reduced TET enzyme activity are associated with a hypermethylated stem-like cellular phenotype linked to glioma aggressiveness [3,4,12]. Overall, loss of the epigenetic 5hmC mark has been associated with increased aggressiveness in GB cell lines [12], while heterogeneity in the expression of 5hmC and TET proteins has been observed across GB tissues and blood samples [13]. In vitro studies demonstrate that high levels of TET2 expression in glioma stem cells (GSCs) are associated with chemoresistance, possibly through the alteration of DNA damage response and repair pathways [12,14,15]. It has been shown in GB tissues and cell models that miR-10b-5p-mediated suppression of TET2 promotes PD-L1-mediated immune evasion [12,16]. A deeper understanding of the role of the TET enzymes in gliomas may contribute to the identification of biomarkers and therapeutic strategies in order to modulate epigenetic reprogramming [3,4,12].

In this review, we compiled current knowledge regarding the structure and function of TET proteins in glioma pathobiology, investigating their impact in the epigenetic mechanisms that contribute to tumor onset and progression [4,17,18]. Moreover, we critically evaluate the diagnostic and prognostic value of TET protein expression and 5hmC levels in gliomas [19,20,21] as well as the emerging therapeutic strategies targeting TET-related pathways, aiming at reversing the malignant phenotype [6,22,23].

## 2. Literature Search and Study Selection

The literature search for this narrative review was performed between May and June 2026, primarily using PubMed, along with Google Scholar used as a supplementary search tool, to identify additional relevant publications without following a formal systematic-review protocol. The search combined terms relating to TET biology, DNA hydroxymethylation, and glioma, including combinations of the following keywords: “TET proteins”, “TET1”, “TET2”, “TET3”, “ten-eleven translocation”, “glioma”, “glioblastoma”, “DNA hydroxymethylation”, “5hmC”, “5-methylcytosine”, “IDH mutation”, “2-hydroxyglutarate”, “epigenetic regulation”, “tumor microenvironment”, “biomarker”, “immunotherapy”, and “epigenetic therapy”. Boolean combinations of these terms were used to identify the literature addressing the major biological and clinical themes covered in the review.

Articles from the preceding five years were preferentially considered to capture recent developments in the field, with earlier seminal studies retained if they provided essential mechanistic and clinical understanding of TET dysregulation in gliomas.

Studies were selected based on their relevance to the objectives of the review, with particular emphasis given on primary human studies, mechanistic investigations, relevant in vitro and in vivo experimental models, and translational studies addressing TET activity, 5hmC biology, IDH-associated metabolic regulation, tumor microenvironment interactions, biomarkers, and therapeutic strategies in gliomas. Review articles and other secondary sources were used primarily to provide conceptual background and to identify relevant primary literature. The studies were qualitatively prioritized according to their methodological rigor, direct relevance to glioma biology and experimental or clinical evidence.

## 3. Epigenetic Landscape in Gliomas

Gliomas are characterized by genetic alterations and epigenetic modifications associated with proliferation, cellular growth, survival and apoptosis, which influence progression of disease, prognosis and therapeutic response [24]. Epigenetic alterations have a significant contribution to the definition of glioma subgroups based on the current World Health Organization (WHO) classification of CNS tumors [25,26]. The main epigenetic mechanisms involved in gliomas include DNA methylation, chromatin remodeling, histone modifications, and regulation through non-coding RNAs (ncRNAs) [27]. These epigenetic modifications are modulated by enzymes called “writers”, “readers” and “erasers” that modify their target by removing, adding, or regulating the interactions between DNA and proteins [28,29].

**Figure 1:**
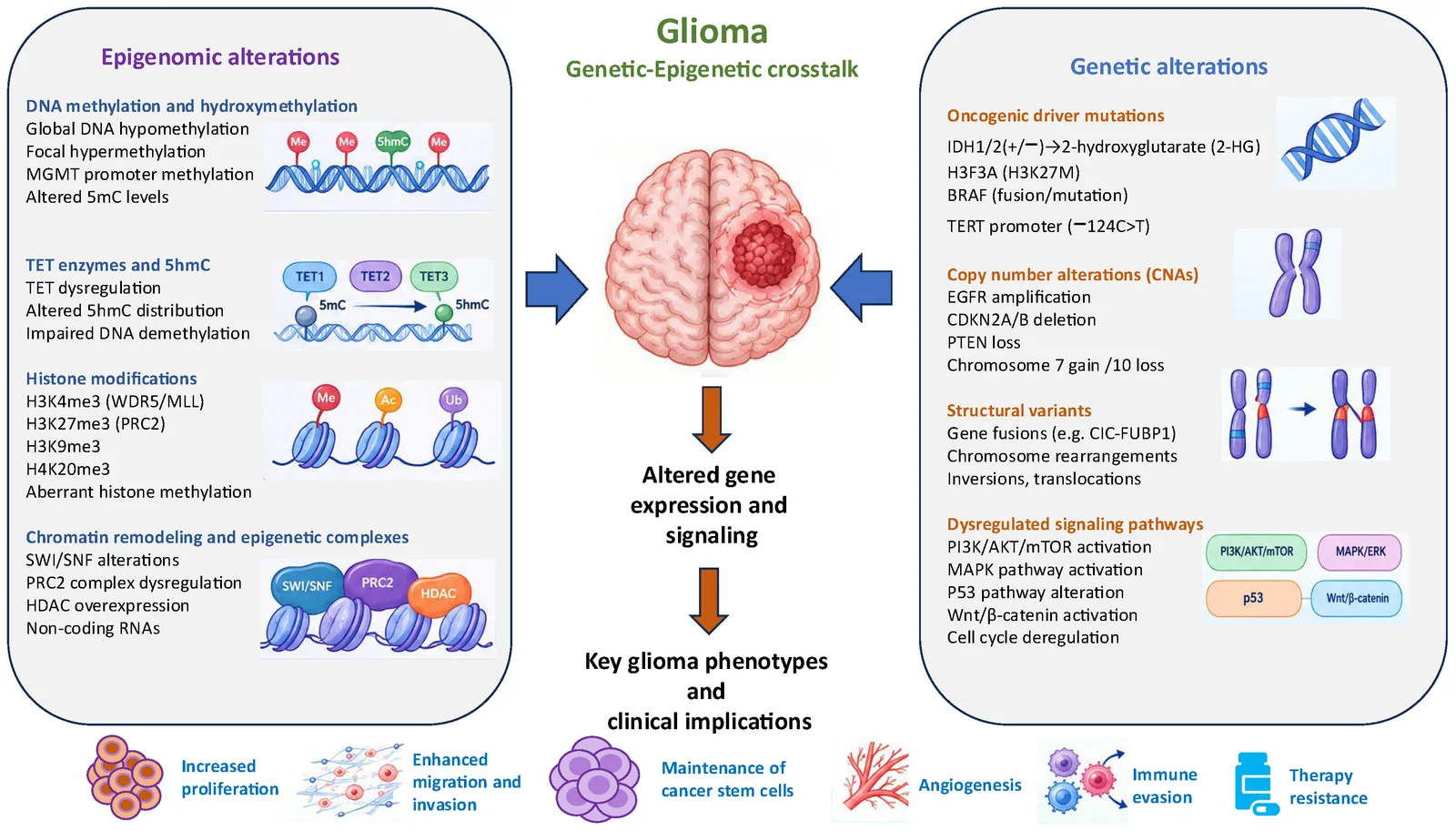
Epigenomic and genetic alterations contributing to glioma development and progression. Schematic representation of the major epigenomic and genetic alterations implicated in glioma biology. Epigenomic alterations include aberrant DNA methylation and hydroxymethylation, dysregulation of TET enzymes and 5hmC, altered histone modifications, and changes in chromatin remodeling and epigenetic regulatory complexes. Genetic alterations comprise oncogenic driver mutations, copy-number alterations, and structural variants, which can affect key signaling pathways involved in glioma biology. Genetic and epigenomic alterations interact and converge to regulate tumor-cell proliferation, migration and invasion, stem-like cell properties, angiogenesis, immune evasion, and therapeutic resistance, ultimately contributing to glioma progression.

To date, DNA methylation is perhaps the best-studied and one of the most stable mechanisms for the transmission of epigenetic information during cell division maintaining gene expression patterns. In mammals, methylation mainly takes place at the 5th position of the cytosine ring, creating 5mC and is usually observed at sites where a cytosine is followed by a guanine, forming the so-called CpG islands [30]. The addition of methyl groups to cytosine residues is mediated by proteins of the DNA methyltransferase (DNMT) family, whereas active DNA demethylation is primarily mediated by proteins of the TET family [19,31]. The initial DNA methylation patterns are established by the de novo DNA methyltransferases, primarily DNMT3A and DNMT3B. Subsequently, during DNA replication and cell division, these methylation patterns are maintained in daughter cells by the DNA methyltransferase DNMT1 (Figure 1) [32].

Extended DNA methylation via the hypermethylation of tumor suppressor genes and the hypomethylation of oncogenes may constitute an initiating factor in oncogenesis [33]. The dynamic balance between DNA methylation and active DNA demethylation ensures tight epigenetic regulation. However, passive DNA demethylation may also occur in the absence or inhibition of DNMT1 and ubiquitin-like with PHD and RING finger domains 1 (UHRF1) or as a result of defects in the DNA methylation maintenance machinery [19]. For example, during DNA replication, passive DNA demethylation can occur when the maintenance methylation machinery fails to efficiently copy methylation marks onto the newly synthesized DNA strand. Although DNMT1 and its targeting factor UHRF1 preferentially recognize hemi-methylated CpG sites containing 5mC, they exhibit substantially reduced activity toward oxidized 5mC derivatives, particularly 5hmC, 5fC, and 5caC. In consequence, these oxidized cytosine modifications can be progressively diluted during successive rounds of DNA replication, with 5hmC being more susceptible to replication-dependent passive loss [34]. Previous studies indicate that DNMTs are overexpressed in gliomas [35]. Emerging studies show that high-grade gliomas present global DNA hypomethylation due to loss of the methylation machinery [36]. This discrepancy brings to the surface the study of DNMT and TET expression in gliomas, especially in the context of IDH mutation status, which affects the epigenetic landscape of gliomas through the production of D-2-hydroxyglutarate (D-2-HG) [37]. IDH-mutant gliomas usually exhibit a hypermethylation phenotype (G-CIMP), whereas IDH-wildtype gliomas lack the G-CIMP phenotype showing different DNA methylation patterns [8,38].

Post-translational modifications (PTMs) of histones also represent an important level of epigenetic modification in gliomas. Histone modifications are reversible changes of chromatin structure and regulate transcription factors accessibility to genomic DNA, affecting gene expression. The most extensively studied modifications include acetylation, methylation, phosphorylation, ubiquitination, succinylation, lactylation, glycosylation and SUMOylation, each of which contributes to the formation of euchromatin or heterochromatin [39]. For example, an immunohistochemical study of tumor samples from 230 glioma patients, who had undergone surgical resection, demonstrated that histone modifications such as H3K4me2, H4K20me3, H3K9Ac and H3K18Ac may have an impact on overall survival (OS) and progression-free survival (PFS) in patients with glioma [40]. An additional analysis using the Oncomine database, which contains numerous published microarray datasets, showed that histone deacetylases (HDACs) 1,3,6,9 are overexpressed in brain malignancies, indicating a critical role for certain HDACs in active glioma tumorigenesis [41]. Another study showed that the self-renewal capacity of GB cancer stem cells (CSCs) and tumor-initiating ability in vivo were significantly reduced upon targeted pharmacological disruption of the activity of Enhancer of Zeste Homolog 2 (EZH2), a histone methyltransferase that constitutes the catalytic subunit of the epigenetic complex Polycomb Repressive Complex 2 (PRC2) [42].

Chromatin remodeling represents a significant factor of chromatin modification accessibility via ATP-dependent repositioning, eviction or restructuring of nucleosomes [43]. Particular attention has been given to the protein Alpha Thalassemia/Mental Retardation Syndrome X-linked (ATRX), which is involved in nucleosome remodeling, heterochromatin maintenance, DNA replication and telomere stability [44]. Loss-of-function mutations of ATRX frequently occur in grade 2,3 astrocytomas (71%), oligoastrocytomas (68%) and secondary GB (57%), being also associated with IDH1 mutations [45]. Furthermore, loss-of-function mutations of ATRX have been associated with Alternative Lengthening of Telomeres (ALT) in IDH-mutant gliomas, a mechanism that contributes to gliomagenesis [46]. The chromatin remodeling complex, Switch/Sucrose Non-Fermentable family (SWI/SNF) has demonstrated increasing preclinical evidence which supports its essential role in maintaining the stemness of GSCs [47]. Recently, ncRNAs, including microRNAs (miRNAs), long non-coding RNAs (lncRNAs) and circular RNAs (circRNAs), have emerged as important epigenetic regulators in gliomas, contributing to the regulation of gene expression and influencing fundamental cellular processes as well as the maintenance of glioma stem cells, contributing to the malignant phenotype [48,49,50].

The epigenetic mechanisms described above represent a network that regulates gene expression [51]. The reversible nature of epigenetic modifications has attracted current research interest, as their plasticity may provide opportunities for developing therapeutic approaches in gliomas [52]. Among the currently established biomarkers, O6-methylguanine-DNA methyltransferase (*MGMT*) promoter methylation represents one of the most clinically relevant epigenetic alterations [53]. Inhibitors targeting DNMTs, histone deacetylases (HDACs), histone methyltransferases, as well as enzymes involved in active DNA demethylation, such as TET proteins, are currently under extensive investigation [5,54,55].

## 4. The TET Protein Family: Structure and Function

The TET proteins (TET1, TET2 and TET3) belong to the Fe(II)/α-KG-dependent dioxygenases that mediate DNA demethylation and serve as regulators of epigenetic programming. In addition to their catalytic activity, they are implicated in chromatin remodeling, transcriptional control and the maintenance of cellular phenotype across physiological and pathological contexts [32,56,57].

**Figure 2:**
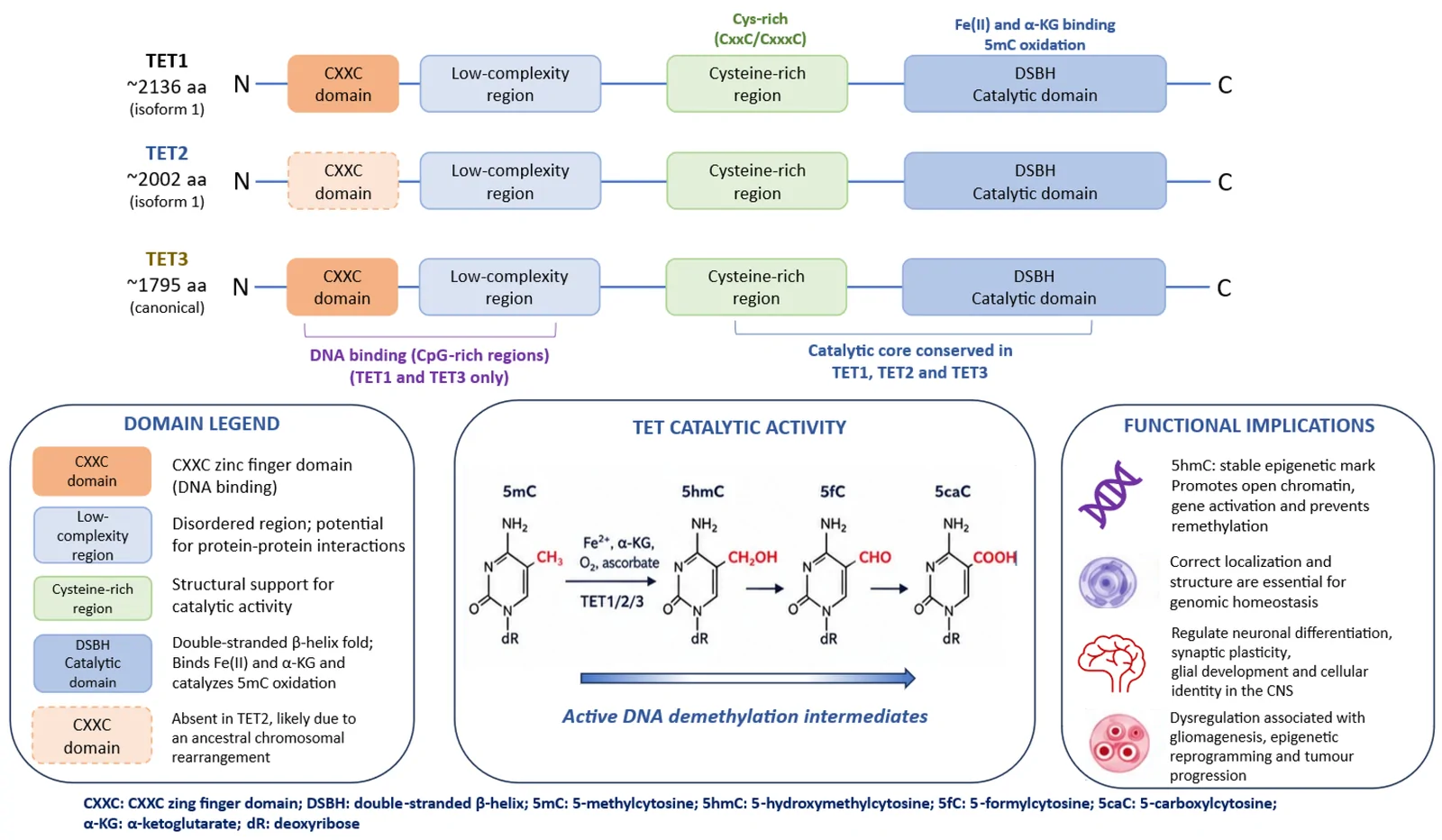
Structural organization and catalytic mechanism of mammalian TET proteins. Schematic representation of the domain organization of the three mammalian ten-eleven translocation (TET) DNA dioxygenases, TET1, TET2, and TET3, and their Fe(II)/α-ketoglutarate (α-KG)-dependent catalytic activity. TET1 and TET3 contain an N-terminal CXXC zinc-finger domain implicated in DNA binding, whereas the CXXC domain is absent from TET2. The proteins also contain variable low-complexity regions, cysteine-rich regions that support the catalytic architecture, and a conserved double-stranded β-helix (DSBH) catalytic domain responsible for 5-methylcytosine (5mC) oxidation. The catalytic core contains the conserved Fe(II)- and α-KG-binding sites and catalyzes the sequential oxidation of 5mC to 5-hydroxymethylcytosine (5hmC), 5-formylcytosine (5fC), and 5-carboxylcytosine (5caC), in the presence of molecular oxygen and appropriate reducing equivalents such as ascorbate. The resulting oxidized cytosine derivatives can contribute to active and passive DNA demethylation and epigenetic reprogramming.

Structurally, all TET proteins share a highly conserved C-terminal catalytic region that contains a cysteine-rich domain followed by a double-stranded β-helix (DSBH) fold, also known as the jelly-roll domain, which belongs to the family of Fe(II)/α-KG-dependent dioxygenases (Figure 2). This catalytic core coordinates Fe(II) and α-KG through conserved histidine and aspartate residues, enabling the sequential oxidation of 5mC into 5hmC, 5fC, and 5caC, thus initiating active DNA demethylation [58]. The cysteine-rich region contributes to substrate recognition, catalytic efficiency, and stabilization of the enzyme-DNA complex, whereas the DSBH domain harbors the catalytic active site responsible for oxidation reactions. TET1 and TET3 proteins can be distinguished from TET2 by the presence of an N-terminal CXXC-type zinc finger domain required for DNA binding in CpG-rich regions. In contrast, TET2 lacks an intrinsic CXXC domain due to an ancestral chromosomal inversion that separated the catalytic gene from its original CXXC-encoding region, giving rise to the independently expressed CXXC4 (*IDAX*) gene [32,56]. Rather than functioning as a DNA-binding module for TET2, IDAX interacts with TET2 and regulates its protein stability by promoting caspase-dependent degradation, thereby providing an additional layer of control over TET2 activity. Beyond these conserved domains, the N-terminal regions of TET proteins are intrinsically disordered and contain multiple regulatory motifs that mediate interactions with transcription factors, chromatin-remodeling complexes, histone modifiers and post-translational modification enzymes (Figure 2) [4,17,18].

These structural differences confer distinct DNA-binding properties, chromatin occupancy profiles, regulatory interactions, and tissue-specific biological functions to the three TET isoforms, explaining their non-redundant roles in embryonic development, lineage specification, stem cell maintenance, and disease pathogenesis. In the CNS, TET proteins are highly expressed in neural progenitors and adult neurons and 5hmC is one of the most abundant epigenetic modifications. This abundance has been highlighted in epigenetic studies showing that 5hmC enrichment localized to gene bodies and promoters of neurologically active genes appears as a stable epigenetic mark distinct from 5mC, which contributes to the maintenance of neuronal function [32,56]. TET1 and TET3 regulate genes involved in synaptic plasticity, memory and neuronal differentiation, while TET2 regulates genes involved in glial cell development and response to injury.

Aberrant expression and activity of TET proteins has been associated with cancer onset and progression in several solid tumors, including gliomas. Downregulation or mislocalization of TET1 and TET3 is associated with reduced global levels of 5hmC and increased methylation of the promoter regions of tumor-suppressive genes, promoting a dedifferentiated stem-like state [17,59,60]. It has been demonstrated in mouse models that TET1-mediated hydroxymethylation is indispensable for the activation of genes required for myelination. TET1 loss can effectively impair these neuronal differentiation pathways and may contribute to the malignant phenotype and the chemoresistance observed in high-grade gliomas [14,56,57,59,61].

Phosphorylation, acetylation and O-GlcNAcylation influence TET stability and chromatin association. It has been demonstrated that the integrin α6-STAT3 axis directly upregulates TET3, which maintains the expression of key stemness genes and supports tumor growth [57]. Downregulation of TET1 has been shown to activate the Wnt/β-catenin signaling pathway promoting cell proliferation and invasion [62], while *TET2* promoter methylation has been identified as a distinct epigenetic event characterizing IDH-wild-type low-grade gliomas [63].

## 5. Role of TET Proteins in Glioma Biology

DNA methylation dynamics is an epigenetic mechanism regulated by the enzymes of the TET protein family. Within the physiological range, TET proteins are responsible for the sequential oxidation of 5mC to 5hmC [3,8,18,64]. The latter modification plays an important role in healthy human brains, where the level of 5hmC is high and serves as a key regulator of gene expression in neurons [18]. In turn, gliomas exhibit a specific genome-wide depletion of this epigenetic marker, predominantly affecting single-copy DNA and not repetitive genomic regions. This alteration is associated with an abnormal pattern of DNA hypermethylation targeting enhancers and actively transcribed sites [65]. These genome-wide epigenetic changes have also been associated with tumor aggressiveness, invasion, and poorer patient prognosis [66].

The effects of TET isoforms can vary depending on the cellular and molecular context of glioma development and progression. In IDH-wild-type gliomas, nuclear exclusion of TET1 has been associated with reduced 5hmC levels, indicating that altered TET1 localization may contribute to disruption of epigenetic homeostasis [60]. Moreover, TET1 expression was downregulated in human malignant glioma tissues, and experimental TET1 inhibition was shown to promote glioma cell proliferation, migration, and invasion through activation of the Wnt/β-catenin pathway. In vivo rescue experiments further supported a role for TET1 in regulating the Wnt/β-catenin pathway in glioma cells [62]. However, contrasting findings have shown increased TET1 levels in primary GB cells associating with increased 5hmC levels, suggesting potential variations in cell culture conditions and cell types [67].

Furthermore, it has been shown that ectopic expression of EGFR can suppress TET1 expression in GB cell models, while inhibition of EGFR with gefitinib, as well as inhibition of MEK with U0126, lead to the re-expression of TET1. Inhibition of EGFR was also sufficient to induce the re-expression of four tumor suppressor genes that had been epigenetically silenced through the EGFR pathway. In addition, *TET1* knockdown in U87/EGFRvIII cells prevented the re-expression of the tumor suppressor genes following gefitinib treatment, indicating that the oncogenic activity of EGFR may promote the epigenetic silencing of tumor suppressor genes through a TET1-dependent mechanism [68]. In turn, TET1 has been shown to contribute to the DNA damage response (DDR) in human glial cells. TET1 deficiency in vitro was associated with reduced levels of DNA-PK, which participates in the non-homologous end joining (NHEJ) pathway, as well as with reduced formation of 53BP1 foci following DNA damage induced by irradiation. Therefore, the contributory role of TET1 in DNA repair may indicate that TET1 loss is possibly linked to genomic instability in glial cells [69].

In addition, TET2 has been reported to restrain glioma cell proliferation and invasion, with ectopic TET2 expression significantly inhibiting tumor growth in human glioma xenografts. ZEB1 expression was upregulated in primary glioma tissues and higher levels observed in high-grade compared with low-grade gliomas, and was associated with tumor progression and poorer overall survival. In contrast, TET2 expression was reduced in glioma tissues and inversely correlated with ZEB1 expression. ZEB1 was shown to bind to the *TET2* promoter, while *ZEB1* knockdown increased TET2 expression and *TET2* promoter activity, supporting a role for ZEB1-mediated repression of TET2 in glioma progression [66]. Furthermore, TET2 has been implicated in regulating the interaction between glioma cells and the immune microenvironment, since reduced TET2 expression was associated with increased Programmed Death-Ligand 1 (PD-L1) expression and glioblastoma progression [16].

In contrast to the predominantly tumor-suppressive functions described for TET1 and TET2, the role of TET3 appears to be highly context- and cellular-state-dependent rather than uniformly oncogenic. TET3 levels are significantly higher in tumorigenic GSCs compared with differentiated glioma cells. This increase in TET3 levels occurs through extracellular matrix factors, since the binding of laminin to integrin α6 receptors activates the focal adhesion kinase (FAK) and Signal Transducer and Activator of Transcription 3 (STAT3) pathways, leading to direct induction of TET3 expression. The induction of TET3 results in TET3 complex formation with STAT3, leading to hydroxymethylation of the promoters of various pro-survival factors and oncogenes, including *c-Myc*, *Bcl-XL* and survivin. The 5hmC generated through TET3 activity enables GSC maintenance, self-renewal and further therapy resistance [57].

Importantly, TET3 may also exert tumor-suppressive effects in other glioma cellular contexts. Carella et al. demonstrated that epigenetic repression of TET3 reduces genome-wide 5hmC levels and promotes GB progression, indicating that loss of TET3 can contribute to malignant epigenetic remodeling [62]. These opposing observations may reflect differences between GSC-enriched niches characterized by active integrin α6–STAT3 signaling and more differentiated tumor compartments, in which distinct transcriptional programs may determine the consequences of TET3 activity. Differential promoter usage or alternative splicing may further contribute to functional heterogeneity of TET3 across glial lineages, although this possibility requires further investigation. Thus, TET3 should be viewed as a context-dependent regulator whose effects may vary according to cellular state, microenvironmental signaling and epigenetic context, rather than as exclusively oncogenic or tumor-suppressive.

Recent research demonstrates the non-catalytic roles of TET family by showing that these enzymes can also interact with other epigenetic factors to become transcriptional co-activators or co-repressors, as summarized in Table 1. Therefore, the simultaneous disruption of catalytic and non-catalytic roles of TET enzymes is one of the key molecular players in glioma progression and contributes to drug resistance and immune escape [18].

## 6. TET Protein Effects on Tumor Microenvironment

The tumor microenvironment (TME) is a very complex network of macromolecules, including extracellular matrix (ECM) components, immune cell infiltration and metabolic stresses that can influence tumor cell behavior. In this dynamic environment, TET enzymes can respond to TME changes and contribute to the regulation of the epigenetic state of tumor cells [71].

It was shown in glioma stem cell lines that activation of the laminin–integrin α6–FAK pathway increased STAT3 expression and activation, which directly promoted TET3 expression and increased 5hmC levels. The authors delineated an intracellular signaling axis consisting of ECM–laminin/integrin α6–FAK–STAT3–TET3–5hmC, which contributes to the upregulation of genes critical for the maintenance, survival and proliferation of GSCs, promoting their phenotype and resistance to therapies. In vivo, *TET3* silencing reduced 5hmC accumulation, leading to inhibition of tumor growth. In addition, in the majority of 19 glioma tumor sections, integrin α6 expression was associated with TET3 protein expression [57]. Moreover, hypoxic conditions in a human GB cell line were shown to induce the expression of TET1 and TET3 and their direct binding to regulatory regions of the *OCT4* and *NANOG* genes, contributing to the upregulation of a pluripotency network [72].

It has been further demonstrated in GB tissue samples that increased levels of miR-10b-5p were correlated with decreased TET2 expression. Inhibition of miR-10b-5p or overexpression of TET2 in GB cell models reduced cell viability, migration, and invasion, as well as PD-L1 expression. However, simultaneous inhibition of TET2 with siRNA in the miR-10b-5p inhibition model did not cause a corresponding change in T-cell cytotoxicity, indicating that the effect of miR-10b-5p inhibition on the GB-T cell interaction depends on TET2. Mechanistically, TET2 overexpression or inhibition of miR-10b-5p increased the binding of TET2 and HDAC1/2 to the *PD-L1* promoter, indicating that TET2 recruits HDACs and suppresses *PD-L1* transcription. However, further studies in more cell lines and experimental GB xenograft models are required to confirm the regulatory mechanism and the functional role of the TET2/PD-L1 axis in antitumor immunity [16].

Recent data have demonstrated the NSUN5/TET2/RBFOX2 epitranscriptomic axis plays a crucial role in the regulation of macrophage phagocytosis through the control of CTNNB1 chromatin-associated β-catenin RNA (caRNA) stability and the immune checkpoint CD47 expression. Specifically, in glioma cell lines the canonical 28SrRNA methyltransferase NSUN5 deposits 5mC on CTNNB1 caRNA and subsequently, oxidized to 5hmC in a TET2-dependent manner. The 5hmC-modified caRNA is then recognized by RBFOX2, promoting its degradation and consequently reducing β-catenin and CD47 expression. This has led to enhanced phagocytosis of glioma cells by tumor-associated macrophages (TAMs) in an in vitro co-culture model [73].

Additionally, in vitro and in vivo studies showed that in IDH1/2 mutant gliomas, accumulation of the oncometabolite 2-HG acts as a competitive inhibitor of multiple α-KG-dependent dioxygenases, including histone demethylases and the TET family, thereby impairing TET2 activity [5]. Consequently, an important scientific question is how IDH mutations affect the NSUN5/TET2/RBFOX2 epitranscriptomic axis. In this context, bioinformatic analysis revealed a negative correlation between *NSUN5* gene expression and IDH mutation, while samples from IDH1-R132H-mutant patients confirmed low NSUN5 expression. In vitro experiments show that IDH1-R132H glioma cells required higher dose of RRx-001 (a DNMT1 inhibitor that can epigenetically downregulate NSUN5) compared to WT glioma cells. Further in vitro and in vivo experiments in IDH1-R132H glioma, showed that the combination of ivosidenib (an inhibitor of mutant IDH1) with RRx-001 synergistically increased NSUN5 and downregulated β-catenin and CD47 expression with increased macrophage infiltration in vivo. This combination strategy may represent a potential therapeutic opportunity for IDH1-R132H-mutant gliomas that warrants further investigation [73].

TET/5hmC-dependent epigenetic regulation should also be considered within the broader signaling landscape of gliomas. Phosphoinositide-specific phospholipases C (PI-PLCs), particularly nuclear PLC isoforms, participate in signaling networks regulating cell proliferation, differentiation, cell-cycle progression, and gene expression. Studies investigating the nuclear PI-PLC signaling have identified altered expression or activity of specific PLC isoforms in brain disorders and have suggested that some PI-PLC subtypes may represent molecular signatures of high-grade glioma [74,75]. However, a direct mechanistic relationship between PI-PLC signaling and TET-mediated 5hmC regulation has not yet been established and further studies are required.

## 7. Potential Impact of TET Alterations on Immunotherapy Response

It is well established that IDH1 and IDH2 mutations lead to the pathological accumulation of D-2-HG, which acts as the primary driver of epigenetic alterations. These alterations occur through direct inhibition of TET enzymes by D-2-HG, which induces the global hypermethylation phenotype, G-CIMP. Specifically, the TET enzyme suppression alters the tumor microenvironment and turns it into an immunologically inactive state by disrupting critical inflammatory transcription factors like STAT1 and suppressing key interferon-γ-inducible chemokines such as CXCL10. This chemokine deficiency drastically interrupts the production and development of cytotoxic CD8+ tumor-infiltrating lymphocytes (TILs), which play a key role in the host’s immune response [76].

In order to transition to targeted therapies, it is important to reverse this immunosuppressive state with isoform-selective small-molecule inhibitors of mutant IDH (such as Ivosidenib AG-120). These inhibitors have been developed to suppress D-2-HG production, restore TET enzymatic activity and induce tumor cell differentiation to reverse the D-2-HG effect [6]. In the phase III INDIGO trial, vorasidenib, an oral brain-penetrant dual inhibitor of mutant IDH1/2, demonstrated clinical benefit in patients with residual or recurrent grade 2 IDH1/2-mutant glioma who had undergone surgery but had not received prior radiation or chemotherapy. Patients were randomly assigned to vorasidenib or placebo, and the primary endpoint was imaging-based progression-free survival, which was significantly improved with vorasidenib compared with placebo (27.7 vs. 11.1 months; HR, 0.39; *p* < 0.001), with a significant delay in the time to the next anticancer intervention also observed [77]. These findings established vorasidenib as an effective targeted therapy for this selected population indicating the need for further evaluation across broader clinical settings.

However, preclinical evidence highlights a major therapeutic obstacle, which indicates that the global DNA hypermethylation can persist even after mutant IDH is pharmacologically reduced and D-2-HG levels return to normal, a phenomenon that can be characterized as epigenetic memory [6]. Mechanistically, prolonged TET inhibition may establish a stable epigenetic state through the recruitment of repressive chromatin modifiers and maintenance methylation machinery, including H3K9me3/H3K27me3 and DNMT1/UHRF1, thereby allowing aberrant methylation patterns to persist independently of D-2-HG. Persistent DNA hypermethylation may also disrupt CCCTC-binding factor (CTCF)-dependent chromatin boundaries and Topologically Associating Domain (TAD) organization, permitting aberrant enhancer-promoter interactions that sustain oncogenic transcription, providing a rationale for combining IDH inhibitors with DNA hypomethylating or chromatin-modifying therapies. Combination therapies, where IDH inhibitors are used together with immunomodulatory agents, have attracted increased research activity [77]. Studies of in vivo glioma models, combining IDH inhibitors (like Ivosidenib) with small immunomodulatory molecules (like RRx-001) which target CD47/SIRpa), lead to the NSUN5/TET2-directed RNA modification pathway, enhance macrophage-mediated phagocytosis and reverse glioma immune evasion [73].

Recently, there has been a focus on strategies aimed at enhancing TET activity through the use of vitamin C (ascorbate) as a cofactor of TET enzymes. In a cohort of 37 glioma patients, of whom 11 had been diagnosed with low-grade glioma (LGG, grades 2,3) and 26 with GB, a reduced ascorbate content was observed in high-grade gliomas (HGG) compared with LGG, as well as a positive correlation between ascorbate levels and global 5hmC levels. In addition, there was no positive correlation between ascorbate levels and the methylation status of the *MGMT* promoter, which may be explained by the fact that *MGMT* promoter methylation may be regulated independently of global changes in 5hmC levels. These data have already paved the way for clinical studies which evaluate the adjuvant therapeutic effects of ascorbate in patients with glioma [78]. However, further studies in larger cohorts are required for increased statistical power and taking into account the factor of glioma heterogeneity.

## 8. Methods for Assessing TET Activity and 5hmC Levels in Clinical Samples

The importance of 5hmC levels and TET activity as significant factors contributing to the pathogenesis of gliomas has been revealed [79]. LC-MS and immunohistochemistry have shown that the levels of 5hmC are highest in neuronal cells of the CNS compared with other tissues in mice [80]. Of note, the distribution of 5hmC and 5mC levels differs in the mouse genome, with 5hmC being predominantly located at enhancers, promoter CpG islands (CGIs) and intragenic regions (“bodies”) of genes [81]. Furthermore, studies have shown that the levels of 5mC and 5hmC differ among different cell types in the human brain and 5hmC is also being investigated as a potential biomarker for aggressive tumor phenotypes associated with cancer stem cell phenotype and cancer progression [57,82,83,84]. Therefore, complementary methods that assess DNA methylation levels have attracted research interest. These technologies have evolved from methods that assess global methylation to high-resolution techniques that allow the analysis of specific CpG sites, while novel bisulfite-free approaches are being developed to overcome the limitations of existing methods and enable more accurate discrimination between 5mC and 5hmC [85].

Global 5mC and 5hmC abundance can be quantitatively determined using high-performance liquid chromatography with mass spectrometric detection (HPLC-MS) [85,86]. HPLC-MS/MS typically requires 50–100 ng of DNA, although smaller amounts, as low as 5 ng, have also been reported, while an important advantage of this method is that it is not affected by the quality of the DNA sample [87,88]. HPLC-MS is one of the most accurate and sensitive methods for the quantitative determination of total 5hmC in biological samples, but it is technically complex and requires expensive and specialized equipment. In addition, HPLC-MS does not provide information on the genomic distribution of 5hmC at specific genomic loci, limiting its utility in clinical analysis, where rapid and locus-specific assessment of changes in 5hmC is required [89].

Locus-specific 5hmC mapping can be performed using hydroxymethylated DNA immunoprecipitation (hMeDIP)- based approaches and other enrichment strategies, such as J-binding protein 1 (JBP1) and sequential glycosylation, oxidation and biotinylation (GLIB)-based methods [90,91,92]. Regarding single-base resolution profiling, conventional bisulfite sequencing (BS-seq) cannot distinguish 5hmC from 5mC. Additionally, the standard Infinium methylation arrays cannot independently distinguish 5mC from 5hmC following conventional bisulfite treatment, unless combined with an appropriate chemical or enzymatic strategy, such as oxidative bisulfite treatment [93,94]. This limitation has been overcome by Tet assisted bisulfite sequencing (TAB-seq) and oxidative bisulfite sequencing (oxBS-seq) that enable the discrimination and mapping of 5hmC at individual cytosine sites [95,96]. The evaluation of TET protein catalytic activity can be evaluated through the quantification of the oxidation products of 5mC (5hmC, 5fC, and 5caC) using techniques such as HPLC-MS/MS and the determination of enzymatic kinetic parameters, including catalytic turnover number (kcat) and the Michaelis constant (Km) [97,98]. In addition, TET expression at the transcript level, protein abundance and subcellular localization can be assessed using qRT-PCR, Western blotting and immunohistochemistry or immunofluorescence, respectively [13,14,60,67,99].

However, the interpretation of 5hmC profiles in biological samples can be affected by tissue cellularity and normal brain admixture, as well as by intratumoral heterogeneity [100,101]. Furthermore, DNA degradation in formal infixed, paraffin-embedded (FFPE) samples and batch-related technical variation may affect the reliability and comparability of methylation measurements [102,103]. The sensitivity and specificity of the chosen assay should also be considered, since different 5hmC profiling approaches may produce variable results depending on 5hmC abundance, cell type and genomic context [93].

## 9. Integration of TET Profiling in Molecular Glioma Classification

The latest classification of CNS tumors, especially the revisions of the WHO in 2016 and 2021, has transitioned from the exclusive use of histopathology towards molecular and epigenetic biomarkers with a major component being the identification of IDH1 and IDH2 mutations. Mutant IDH enzymes produce the oncometabolite 2-HG that competitively inhibits α-ketoglutarate-dependent dioxygenases, including TET family enzymes, and leads to widespread epigenetic alterations. Whole-genome DNA methylation arrays (WGMA) have been used for the identification of G-CIMP together with key copy number variations, including 1p/19q codeletion, in a single assay. WGMA has revealed robust epigenetic signatures capable of distinguishing IDH-mutant from IDH-wild-type tumors, particularly facilitating the identification of rare pathogenic IDH variants which may escape detection by standard immunohistochemical methods [104]. However, as mentioned above, standard methylation arrays cannot distinguish 5mC from 5hmC following conventional bisulfite treatment. In the case of specific assessment of 5hmC and 5mC in clinical analysis, specialized chemical or enzymatic preprocessing, such as oxBS or TAB-Seq, is required in order to prevent inaccurate genomic annotation [89].

In a recent study that characterized the profile of 5hmC in human GB cell lines, the loss of 5hmC was negatively correlated with the high WHO grades of GB, while a significant increase in global 5hmC abundance in nearly one-third of GB was observed [13]. This result supports other findings that showed heterogeneity in 5hmC expression throughout the majority of GB [67,105]. The regulation of TET transcription and translation, as well as the nuclear localization of TET, may represent a possible biological explanation for the observed variability [13,30,60]. An attempt has been made to evaluate the levels of TET transcripts as potential biomarkers for the diagnosis of glioma, in which 34 plasma samples from patients with glioma and 5 independent blood samples from healthy volunteers were investigated. In this study, TET1 levels gradually decreased with increasing glioma grade, while TET3 levels remained unchanged and TET2 levels showed a significant increase in grade 2 glioma. However, these findings were obtained from a small number of patients, and their validation in a larger patient population is required [13].

The role of 5hmC in DNA methylation changes in glioma has been further investigated. The global and locus-specific levels of 5hmC and 5mC in primary human glioma samples have been analyzed, using oxBS (12 non-cancerous brains and 53 gliomas). The study showed that, in normal brain cells, 5hmC was enriched in low-CpG-density regions and introns and was associated with histone modifications linked to active chromatin regions, including H3K4me1, H3K36me3, H4K20me1, and H3K79me2. On the other hand, in gliomas, aberrantly hypermethylated CpG sites were identified both with and without loss of 5hmC levels. The previous hypermethylated CpG sites that showed loss of 5hmC were associated with active histone modifications, including H3K4me1, H3K36me3, H3K79me2, H4K20me1, and H3K4me2. Therefore, these CpG sites may have a distinct chromatin signature, compared with the repressive chromatin signature of genes that undergo aberrant hypermethylation in cancer, revealing a possible new type of 5hmC-dependent aberrant DNA hypermethylation that requires further investigation [65].

*TET2* promoter methylation was detected in 5 of 35 (14%) low-grade diffuse gliomas lacking IDH1/2 mutations, whereas it was not detected in any of the 38 IDH1/2-mutant tumors, suggesting that *TET2* promoter methylation may represent an alternative pathogenic mechanism in a small subset of IDH1/2-wild-type gliomas [63]. Already, studies have highlighted that integrated analysis of DNA methylation, gene expression, copy number variation, and mutation data can significantly contribute to further molecular stratification of isocitrate dehydrogenase (IDH)-mutant gliomas [20,104]. It is important that this line of investigation has also been extended to pediatric CNS tumors. In a study of pediatric gliomas, ependymomas, and embryonal tumors, the analysis of 5hmC and 5mC at the nucleotide level showed significantly reduced 5hmC levels in tumors compared with non-cancerous tissues. In addition, the use of OxBS technology, which allows the separation of 5mC from 5hmC, led to changes in the diagnoses that emerged from the existing methylation-based tumor classification systems. This finding reflects both the heterogeneity of tumors at the epigenetic level and the possibility that 5hmC may represent an important parameter for further investigation for its potential integration into the classification of pediatric CNS tumors [106].

**Figure 3:**
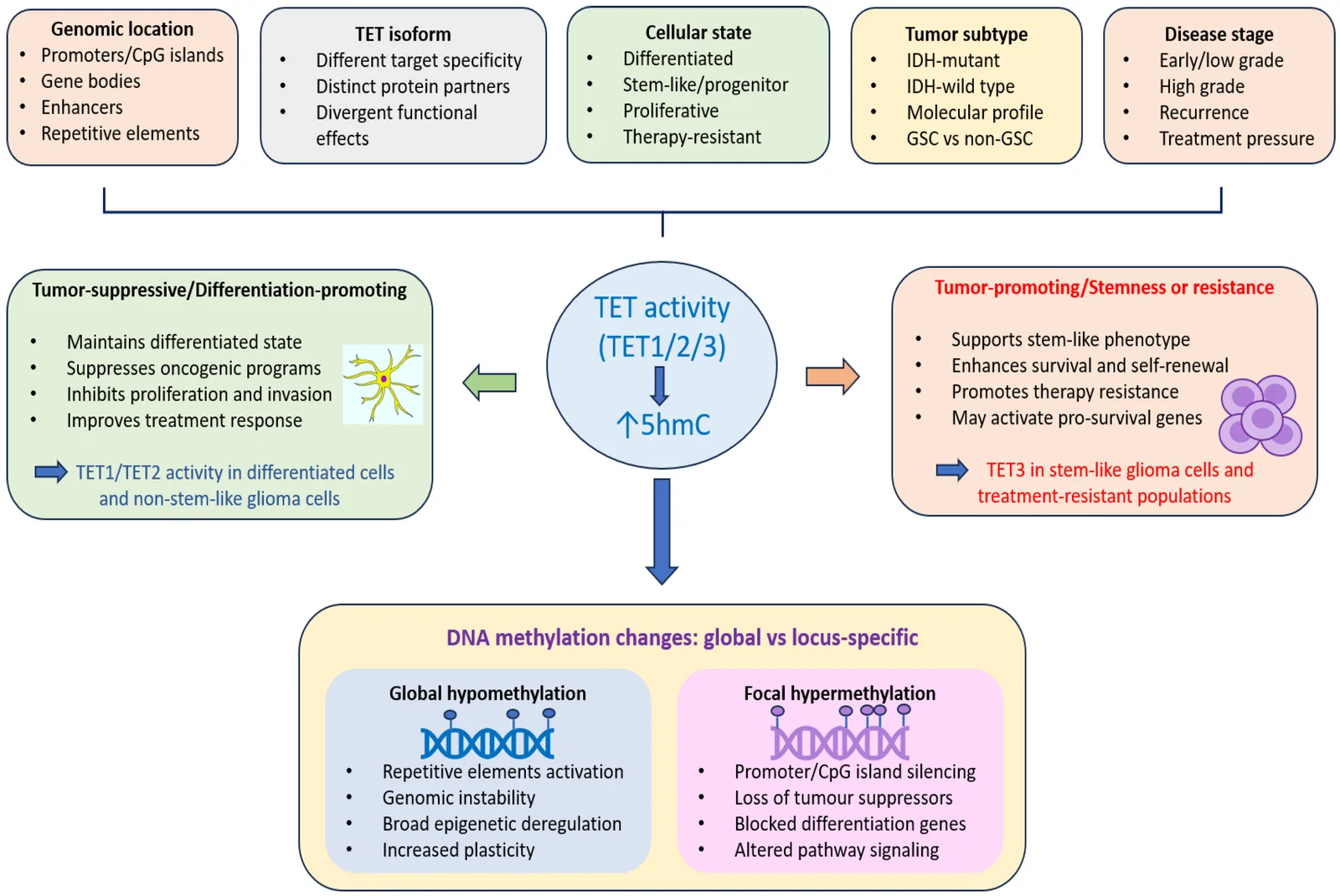
Context-dependent effects of TET activity and DNA methylation in glioma. TET enzymes and DNA methylation should not be considered intrinsically tumor-suppressive or tumor-promoting, as their biological effects depend strongly on the cellular, genomic, and tumor context. TET-mediated oxidation of 5mC and the resulting changes in 5hmC can promote differentiation, restrain oncogenic programs, maintain genomic stability, and enhance therapeutic responsiveness in certain cellular and molecular settings. Conversely, in specific tumor contexts, TET activity and altered DNA methylation may support stem-like properties, cellular plasticity, survival, or resistance to therapy. These divergent effects may reflect differences in TET isoforms, genomic location of the affected loci, cellular state, tumor subtype and molecular background, tumor microenvironment, and disease or treatment stage. Similarly, global DNA hypomethylation and focal or promoter-associated hypermethylation can coexist within the same tumor and have distinct biological consequences.

Taken together, the contradictory observations concerning TET activity and DNA methylation in gliomas may reflect differences in genomic, cellular, and disease context rather than true biological inconsistencies. TET-mediated DNA hydroxymethylation can contribute to maintenance of differentiated cellular states and suppression of tumor-associated epigenetic programs in some settings, whereas the activity of specific TET isoforms may support stem-like phenotypes, survival, or treatment resistance in others. These divergent effects are likely influenced by the TET isoform involved, the genomic regions targeted, the underlying molecular subtype, the differentiation state of the tumor cells, and the stage of disease progression. Similarly, global DNA hypomethylation can coexist with focal hypermethylation of promoters or CpG islands, because these measurements capture different levels of epigenetic organization. Global hypomethylation may affect repetitive elements, genomic stability, and broad regulatory landscapes, while focal hypermethylation can silence specific tumor-suppressive or differentiation-associated genes (Figure 3). Therefore, changes in global 5mC or 5hmC levels should not be interpreted independently of their genomic distribution. A comprehensive understanding of TET-dependent epigenetic regulation in glioma consequently requires integration of global methylation patterns with locus-specific alterations, cellular phenotype, molecular tumor subtype and disease evolution.

## 10. Targeting TET Pathways: Opportunities and Difficulties

Therapeutic targeting of the TET/5hmC regulatory axis represents a promising but complex area in glioma research. The close association between reduced 5hmC levels and glioma cell proliferation, invasion, and treatment resistance suggests that restoration of physiological TET activity may have therapeutic potential [18]. However, most strategies aiming at directly or indirectly modulating TET activity remain at the biochemical, cellular, or preclinical stage and should therefore be considered experimental rather than clinically actionable. One potential approach involves metabolic cofactors such as vitamin C, which can enhance TET catalytic activity by maintaining iron in its reduced state and thereby supporting TET-dependent DNA demethylation. Although this approach provides a mechanistic rationale for restoring TET activity, its therapeutic efficacy and optimal delivery in gliomas remain to be established.

An important strategy for indirectly modulating TET-associated epigenetic dysfunction involves targeting mutant IDH1 and IDH2 [22]. Mutant IDH enzymes generate the oncometabolite 2-HG, which can inhibit α-ketoglutarate-dependent dioxygenases, including TET enzymes, thereby contributing to altered DNA methylation. Several mutant-IDH inhibitors have consequently been investigated but these compounds should not be considered interchangeable. Ivosidenib (AG-120) selectively targets mutant IDH1, whereas enasidenib (AG-221) targets mutant IDH2. They have both demonstrated effective suppression of 2-HG in IDH-mutant models and have been investigated clinically in other IDH-mutant malignancies. Vorasidenib is a dual mutant IDH1/IDH2 inhibitor with CNS activity and has provided the strongest clinical evidence to date for IDH-directed therapy in glioma. In the phase 3 INDIGO trial, 331 patients with residual or recurrent, measurable, non-enhancing, IDH1- or IDH2-mutant WHO grade 2 astrocytoma or oligodendroglioma who had undergone surgery without prior chemotherapy or radiotherapy were randomized to vorasidenib 40 mg daily or placebo. Vorasidenib significantly improved imaging-based progression-free survival, the primary endpoint, compared with placebo (median 27.7 vs. 11.1 months; HR 0.39, 95% CI 0.27–0.56; *p* < 0.001), and significantly prolonged time to the next intervention [76]. These findings established a clinically meaningful benefit for vorasidenib in this specific molecular and clinical setting and subsequently supported its regulatory approval for patients aged ≥12 years with susceptible IDH1- or IDH2-mutant grade 2 astrocytoma or oligodendroglioma following surgery.

However, the selected INDIGO population limits extrapolation to higher-grade, previously treated, enhancing, or IDH-wild-type gliomas. Moreover, although reduction of 2-HG provides a strong mechanistic rationale for relieving inhibition of TET and other α-KG-dependent enzymes, clinical evidence that IDH inhibition consistently restores TET activity, reverses established DNA methylation patterns or enhances responses to immunotherapy in patients with glioma remains limited.

Prolonged TET inhibition may also establish a stable epigenetic state through the recruitment of repressive chromatin machinery, including H3K9me3/H3K27me3 and DNMT1/UHRF1-dependent maintenance methylation, allowing aberrant methylation patterns to persist even after D-2-HG levels are normalized. Persistent hypermethylation may further disrupt CTCF occupancy and TAD organization, enabling aberrant enhancer–promoter interactions and providing a mechanistic rationale for combining IDH inhibitors with hypomethylating or chromatin-modifying therapies. Therefore, the clinical benefit of IDH inhibition should not be interpreted as direct evidence that TET restoration is itself sufficient or responsible for the observed therapeutic effect. Furthermore, recent preclinical studies suggest that combining IDH1 inhibition with CD47/SIRPα blockade may enhance TET2- and NSUN5-associated activity and increase macrophage-mediated elimination of glioma cells [73]. Such combinations remain investigational and require further validation in clinically relevant models.

TET-associated alterations in DNA repair also provide a potential basis for indirect therapeutic targeting [6,107]. The impaired DNA repair phenotype associated with mutant IDH and TET dysfunction may create specific vulnerabilities that can be exploited pharmacologically. For example, Artesunate has been investigated in relation to the ALKBH2 DNA repair pathway and may increase the vulnerability of glioma cells with altered DNA repair capacity [107]. Similarly, combining metabolic modulators with targeted epigenetic approaches directed against downstream hypermethylated targets, such as G0S2, has been reported to suppress tumor invasion in vivo [108]. These findings support the concept of exploiting TET-associated molecular vulnerabilities rather than directly manipulating TET enzymes; however, these approaches remain preclinical and require further investigation regarding efficacy, specificity, pharmacodynamics, and safety.

Direct modulation of TET activity is another emerging therapeutic avenue. Beyond metabolic cofactors such as vitamin C, TET expression and stability may potentially be influenced through regulatory non-coding RNA networks. For example, the lncRNA AC016405.3 has been shown to exert tumor-suppressive effects by acting as a competing endogenous RNA for miR-19a-5p, thus reducing repression of TET2 transcripts [15]. In a similar way, PROSER1 has been implicated in the post-translational stabilization of TET2 through O-GlcNAcylation, suggesting that modulation of TET2 protein stability could preserve its enzymatic activity [109]. These observations provide mechanistic evidence for regulation of the TET pathway at the RNA and protein levels, but therapeutic manipulation of these regulatory mechanisms remains experimental.

Conversely, inhibition of TET activity may be relevant in specific biological contexts in which individual TET isoforms contribute to tumor-promoting phenotypes. Cytosine-based compounds such as Bobcat339 were initially reported as competitive inhibitors of TET1/2 catalytic activity and have been widely used as experimental tools to investigate TET-dependent mechanisms. However, subsequent chemical-biological studies have questioned its direct inhibitory activity against purified human TET1 and TET2, showing that inhibition observed with some preparations was attributable to trace copper(II) contamination rather than specific binding to the TET catalytic site. More recent evidence suggests that Bobcat339 may exert its cellular effects, at least in part, through VHL E3 ligase-dependent proteasomal degradation of TET3 rather than through direct catalytic inhibition. Therefore, Bobcat339 should not be considered a validated, selective TET1/2 catalytic inhibitor, and the development of potent, drug-like, isoform-selective TET catalytic inhibitors remains an important unmet need in TET chemical biology [64]. More broadly, the context-dependent functions of individual TET isoforms suggest that future approaches may need to consider selective rather than indiscriminate modulation of TET activity. Hypoxia modulation, DNA-repair sensitization, non-coding RNA-targeted approaches, and selective TET inhibitors therefore represent potential avenues for further preclinical investigation rather than established therapeutic strategies.

Several important barriers currently limit translation of TET-directed approaches into clinical practice [18]. First, the development of pharmacologically suitable TET activators or inhibitors requires adequate isoform selectivity, target engagement, and control of the extent and genomic distribution of TET modulation. Although cytosine-based inhibitors have demonstrated selective inhibition of TET1/TET2 relative to DNMTs in vitro, their pharmacological properties and therapeutic index in glioma remain insufficiently established [64]. Important translational questions to be considered in future studies include BBB penetration, effective target engagement and possible adverse genome-wide effects. Second, the biological functions of TET proteins are highly context- and isoform-dependent [18]. While restoration of TET1/TET2 activity may counteract oncogenic hypermethylation in some settings, TET3 has also been implicated in aggressive glioma stem-like cells, where it can interact with STAT3 and promote hydroxymethylation of pro-survival genes [57]. Thus, the available evidence supports the hypothesis that therapeutic modulation of the TET pathway may need to be isoform- and context-specific rather than uniformly activating or inhibiting TET enzymes. At present, achieving such selective and potentially locus-specific epigenetic modulation remains a major challenge. Accordingly, further pharmacokinetic/pharmacodynamic studies, molecularly defined in vivo models, and prospective clinical investigations are required before TET-directed strategies can be considered clinically actionable in gliomas [18].

Overall, the therapeutic landscape of TET-related targeting should be viewed as an emerging preclinical field. The strongest translational rationale currently derives from approaches that indirectly influence TET-dependent epigenetic states, particularly in molecularly defined IDH-mutant tumors, whereas direct TET modulation, non-coding RNA manipulation, PROSER1 targeting, DNA-repair sensitization, hypoxia modulation, and immune-based combinations remain experimental. A clearer distinction between these evidence levels is essential for evaluating the future therapeutic potential of the TET/5hmC axis in glioma.

## 11. Preclinical Models and Translational Epigenetic Trials in Gliomas

Early mechanistic studies established that TET dysfunction in gliomas can produce widespread alterations in DNA methylation and chromatin organization rather than affecting isolated genes. In particular, the loss of TET-mediated demethylation at CTCF insulator sites can disrupt chromatin insulation and facilitate aberrant enhancer–promoter interactions, including activation of oncogenic loci such as *PDGFRA*, thereby contributing to gliomagenesis [8]. In parallel, molecular studies demonstrated that TET2 expression can be epigenetically suppressed by transcriptional regulators such as ZEB1 and SOX2, promoting an undifferentiated, invasive, stem-like phenotype in glioma cells [12,66].

The development of in vitro and in vivo glioma models that recapitulated the impaired oligodendrocyte differentiation observed in human tumors provided important platforms for investigating the functional consequences of TET dysfunction and for early therapeutic screening [110]. Before the emergence of more selective epigenetic strategies, these models were used to evaluate broad-spectrum DNA hypomethylating agents, including 5-azacytidine and decitabine, with the aim of reversing aberrant DNA hypermethylation. However, the therapeutic potential of global DNA demethylation was limited by its lack of molecular specificity, potential toxicity and inconsistent biological effects. These limitations, together with mixed pathological and therapeutic outcomes, highlighted the challenges associated with targeting the epigenome globally and prompted a transition toward approaches exploiting specific molecular dependencies in TET-deficient gliomas [17].

Early translational studies also suggested that epigenetic alterations could have prognostic and predictive relevance. For example, altered methylation of *HOX* gene clusters and IGFBP2 expression emerged as potentially informative molecular features associated with clinical outcome in glioma patients undergoing epigenetically directed therapeutic approaches [20]. Collectively, these primary studies established the feasibility of manipulating DNA methylation in glioma while simultaneously demonstrating the limitations of nonspecific hypomethylating therapy.

More recent research has shifted from global manipulation of DNA methylation toward the identification of specific vulnerabilities created by TET dysfunction. A key mechanistic advance was the demonstration that impaired TET1 activity can compromise DNA repair pathways in human glial cells, suggesting that TET-deficient tumors may become selectively dependent on alternative DNA damage-response mechanisms [69]. Rather than attempting to restore the global methylation state, this approach provides a rationale for exploiting the accumulated DNA repair stress associated with TET dysfunction as a potential therapeutic vulnerability.

This concept has subsequently encouraged investigation of agents capable of selectively exploiting DNA repair defects in glioma cells. For example, the antimalarial compound, artesunate has been investigated as a potential therapeutic agent that can exploit TET-associated DNA repair deficiencies and promote apoptosis in glioma models. More recent work has further refined this concept by examining specific DNA repair pathways, including ALKBH2-dependent repair mechanisms, as potential synthetic lethal targets in TET-deficient or DNA repair-impaired gliomas [107]. These contemporary approaches are conceptually distinct from broad-spectrum hypomethylating therapy because they seek to exploit a defined molecular dependency generated by the tumor’s epigenetic state rather than inducing nonspecific genome-wide demethylation.

It is evident that the field has progressively evolved from attempts to reverse aberrant DNA methylation globally toward precision strategies that target the downstream consequences of TET dysfunction, including altered chromatin organization, defective DNA repair, and increased dependence on compensatory repair pathways. This transition from epigenome-wide modulation to vulnerability-directed therapy represents an important step toward developing more selective and potentially less toxic therapeutic approaches for molecularly defined glioma subgroups. The progression from foundational models and early hypomethylating strategies to contemporary synthetic-lethal approaches is summarized in Table 2.

## 12. Prospects for Personalized Medicine Based on TET-Related Biomarkers

Personalized medicine involves the development of an individually tailored approach to the diagnosis, prediction and treatment of diseases on the basis of the molecular properties of a patient’s tumor. In the case of gliomas, progress in genomics and epigenomics allowed replacing the conventional classification that is based on histopathological criteria with one that is centered around biomarkers such as IDH mutation status, *MGMT* promoter methylation and DNA methylation patterns [4,13,58,65]. Recently, modifications in the TET family of dioxygenase genes, together with loss of 5hmC, have been considered as promising biomarkers which can provide new possibilities for personalized treatment and prognosis [4,58].

The downregulation of TET1, TET2 or TET3, together with decreased 5hmC levels, has been detected in gliomas and, in several contexts, is associated with increased tumor aggressiveness, proliferation and worse outcome. Thus, loss of nuclear *TET1* localization and lower 5hmC levels have been detected in IDH-wildtype gliomas [60], whereas Carella et al. (2020) [61] demonstrated that epigenetic repression of *TET3* promotes glioblastoma progression through genome-wide loss of DNA hydroxymethylation. However, TET3 should not be considered exclusively tumor-suppressive or oncogenic, as its biological effects appear to depend on the cellular and molecular context. In particular, the increased TET3 activity in integrin α6–FAK/STAT3-enriched GSC populations has been associated with increased 5hmC deposition at stemness-associated regulatory regions and maintenance of self-renewal and therapy resistance [57]. Therefore, TET3 expression or 5hmC levels may have different biological and prognostic implications depending on tumor compartment, differentiation state and underlying transcriptional programs. Comprehensive analyses of glioma tissue samples nevertheless support decreased 5hmC as a common epigenetic feature that may have value as a disease biomarker [13,65]. Therefore, TET-related biomarkers should ideally be interpreted in conjunction with cellular context and other molecular features rather than as isolated indicators of tumor behavior.

Besides the prognostic significance, TET-related biomarkers may also have important predictive value with regard to the choice of therapy. Being directly affected by metabolic alterations caused by IDH1/2 mutations, TET-related biomarkers may serve as potential predictors of therapeutic response to IDH-targeted treatments. Oncometabolite 2-HG competes with α-KG at the catalytic site of dioxygenases, including TET enzymes, which promote DNA hypermethylation [5]. Therefore, restoration of normal epigenetic regulation may become a prospective therapeutic option. Apart from their diagnostic and predictive value, the TET proteins can become a target for therapies. Functional studies indicate the TET2 is repressed by SOX2 that promotes GB stem cells maintenance and tumor growth due to altered DNA methylation [12]. Additionally, the regulation of TET-mediated immune checkpoint pathways has become a promising area of research. Du et al. (2023) [16] showed that decreased TET2 activity increases PD-L1 expression, providing immune evasion in GB, whereas Wu et al. (2024) showed the role of TET2-dependent RNA hydroxymethylation in immune escape mechanisms [73].

The future advances in personalized medicine will require combining TET expression, 5hmC profiling, DNA methylation patterns and multi-omics data with clinical and radiological information. Emerging technologies such as single-cell epigenomics, spatial transcriptomics and artificial intelligence-assisted biomarker discovery will facilitate this approach [9,111]. Although clinical translation remains challenging, current preclinical evidence provides a foundation for completing further mechanistic studies and designing well-controlled clinical trials to evaluate the potential of TET-related biomarkers in precision oncology for glioma patients.

## 13. Conclusions

Current evidence indicates that TET proteins are involved in multiple pathways associated with glioma biology, including epigenetic regulation, cellular and interactions between tumors cells and the surrounding environment. TET enzymes are not limited to the role of DNA demethylation. They participate in many processes that influence the initiation, growth, immune evasion, stemness and therapy resistance of gliomas. Therefore, TET proteins dysregulation can be considered as one of the mechanisms responsible for glioma progression, rather than as an outcome of malignancy. Although TET1-3 have been implicated in the regulation of glioma biology, their effects may vary depending on the cellular and molecular context. Experimental evidence indicates that TET isoforms may exert distinct, and in some cases opposing, effects on glioma cell behavior, depending on factors such as cellular state, microenvironmental signaling, disease context, and experimental model. Recent studies show that changes in metabolism, particularly those provoked by mutations in IDH and accumulation of 2-HG, take place together with changes in epigenetics. Nevertheless, despite pharmacological blocking of mutated IDH, the persistence of abnormal methylation changes indicates that epigenetic imprinting is a major biological barrier to sustained therapeutic effects. The implication of the results is that combined approaches targeting metabolism, DNA methylation, checkpoints and TET pathways are likely to provide a therapeutic benefit. Furthermore, the diagnostic utility of TET proteins and 5hmC as standalone biomarkers appears to be limited. Their combination with molecular classification, methylation profiling, transcriptomic approaches, and emerging liquid biopsy technologies may offer a more comprehensive framework for tumor diagnosis, prognosis and treatment stratification. Despite the progress achieved in this field, there are still many issues concerning the non-catalytic activity of TET proteins, the mechanisms of their specific behavior and their regulation in the glioma microenvironment that remain unresolved and require application of joint multi-omics, single-cell and spatial epigenomic approaches.

## Figures and Tables

**Table 1:** Dysregulation of TET Family Proteins in Gliomas and Their Biological Significance.

TET Alteration	Glioma Context	Direct Molecular Consequence	Functional Phenotype	Major Limitation	Reference
TET1 deficiency	Human glial cells	↓ DNA-PK and ↓ 53BP1 foci following DNA damage	Impaired DNA repair and potential genomic instability	limited number of glioma cell lines	[69]
EGFR → TET1 ↓	U87/EGFRvIII and patient-derived GBM models	↓ TET1 → silencing of tumor-suppressor genes	Tumor-suppressor gene silencing	Global 5mC and 5hmC levels were not directly measured to assess the effect of TET1 repression	[68]
↑ TET2 expression	PDX-derived GB GSCs	↑ 5fC/5caC associated with TET2 expression	TET2 expression correlated with DNA-repair gene expression	GSC heterogeneity and variable TET2 expression may limit generalizability across GSC populations	[14]
SOX2 ↑ → TET2 **↓**	Patient-derived GB GSCs; orthotopic GBM xenografts	↓ TET2 → ↓ 5hmC and ↑ 5mC	↑ GSC self-renewal, stemness, and tumor growth	GB heterogeneity may mask TET2 changes in bulk tumors	[12]
miR-10b-5p ↑ → TET2 ↓	GB cells; human GB tissues	PD-L1 ↑	Migration/invasion ↑; immune evasion ↑	Small human cohort; no in vivo validation	[70]
Epigenetic TET3 ↓	Glioma samples; GBM cell lines; glioma xenograft models	Genome-wide 5hmC ↓	GB growth/tumor formation ↑	Downstream mechanisms not fully defined	[61]
Integrin α6-FAK-STAT3 → TET3 ↑	Human primary GSC lines; orthotopic glioma xenograft models	5hmC ↑	GSC-enriched tumor compartments	Mainly GSC-focused models	[57]

↓ Reduction; ↑ Elevation.

**Table 2:** Representative clinical studies investigating TET proteins and DNA hydroxymethylation in human gliomas.

Study Model	Key Findings	Biological and Clinical Significance	Reference
**Human low-grade diffuse gliomas**	*TET2* promoter hypermethylation detected in IDH-wild-type gliomas.	Suggests epigenetic silencing of *TET2* as an alternative molecular mechanism and potential biomarker.	[63]
**Human glioma tissue samples**	*TET1* nuclear exclusion correlated with reduced global 5hmC levels, especially in IDH-wild-type tumors.	Highlights the role of altered TET1 localization in glioma biology and diagnosis.	[60]
**Human glioma cohort**	Loss of 5hmC defined a subgroup with abnormal DNA hypermethylation patterns.	Identifies 5hmC depletion as a clinically relevant epigenetic hallmark with prognostic potential.	[65]
**High-grade glioma specimens**	Reduced DNA hydroxymethylation compared with normal brain tissue.	Supports the association between 5hmC loss and aggressive tumor behavior.	[79]
**Human glioma tissues**	Identification of subtype-specific TET-related hydroxymethylation profiles.	Suggests TET proteins and 5hmC as candidate diagnostic biomarkers.	[13]
**Clinical glioma cohort**	DNA methylation profiling accurately classified IDH-mutant and IDH-wild-type gliomas.	Demonstrates the clinical utility of epigenomic profiling for glioma classification.	[104]
**Human glioblastoma specimens**	Loss of 5hmC in GB tissue and tumor infiltration zones	Identifies 5hmC loss and intratumoral heterogeneity	[101]
**Integrated molecular analysis of IDH-mutant gliomas**	*HOX* gene hypermethylation and overexpression correlated with poor outcome.	Provides improved molecular risk stratification for IDH-mutant gliomas.	[20]
**Clinical glioma cohort**	DNMT and TET expression correlated with WHO grade and patient survival.	Supports TET family members as prognostic biomarkers and therapeutic targets.	[19]

## Data Availability

No new data were created or analyzed in this study. Data sharing is not applicable to this article.
